# Testing for age-dependent effects of dietary restriction on the strength of condition dependence in ejaculate traits in the guppy (*Poecilia reticulata*)

**DOI:** 10.1098/rsos.230805

**Published:** 2023-08-30

**Authors:** Jonathan P. Evans, Elizabeth J. Turnbull, Rowan A. Lymbery

**Affiliations:** Centre for Evolutionary Biology, School of Biological Sciences, University of Western Australia, Perth 6009, WA, Australia

**Keywords:** sperm competition, resource dependence, resource acquisition, condition dependence

## Abstract

Ejaculates can be costly to produce and depend on an individual's condition, defined as the pool of resources allocated to fitness. A method for assessing condition dependence is to manipulate resource availability and test for a reduction in trait expression. Here, we assess the effects of dietary restriction on two determinants of reproductive fitness in the guppy *Poecilia reticulata*—sperm production and sperm motility. Importantly, we administered dietary restriction at distinct developmental stages to test: (1) whether dietary restriction, when applied exclusively to juveniles, compromised the ejaculates of newly mature males; (2) whether any observed effects of dietary restriction seen in (1) were reversible when fish returned to an unrestricted diet; and (3) whether dietary restriction applied exclusively to adults influenced ejaculates. We found detrimental effects of resource limitation on both traits, and these were consistent across the three developmental stages tested. Furthermore, dietary restriction reduced male body weight, but this was partially reversed when diet-stressed juveniles (i.e. group 2) returned to unrestricted diets. This latter result suggests that diet-stressed males may sacrifice growth in order to maintain their investment in ejaculates. Together these findings underscore the importance of resource acquisition in determining the expression of ejaculate traits.

## 1. Introduction

Sexual selection acts on phenotypes that improve the reproductive success of individuals as they compete for reproductive opportunities [1,2]. A central component of sexual selection theory is that the traits that determine competitive access to mates and/or fertilizations are costly to produce and therefore depend on an individual's condition, defined as the pool of resources that individuals have available to allocate to fitness-enhancing traits [3,4]. Condition therefore depends on how efficient individuals are at both acquiring and allocating resources to competing fitness traits, and only males in high condition will be able to bear the marginal costs of maintaining such traits [5].

As condition depends on patterns of resource acquisition and allocation, and many fitness-enhancing traits are metabolically costly to produce and maintain [6], a useful method for assessing condition dependence is to manipulate resource availability in the form of dietary restriction and test for a reduction in the expression of sexually selected traits. Accordingly, numerous studies have reported that the expression of sexual traits (usually in males) such as ornaments, weapons and behaviours exhibit reduced expression following experimental reductions in food availability [7]. Similarly, studies of post-copulatory sexually selected traits (i.e. those involved in securing competitive fertilizations such as sperm quality, seminal fluids, etc.) have revealed markedly reduced expression of such traits after periods of food limitation [6].

Despite the accumulating evidence that male pre- and post-copulatory sexually selected traits are sensitive to dietary stress, there remain numerous examples that report opposing patterns (e.g. heightened expression under diet limitation [8]) or weak/absent effects of diet on such traits (e.g. [9,10]). Some of this variation may be explained by the type of sexual trait under investigation, as the effects of male condition on trait expression can vary according to the marginal fitness gains associated with investment into specific traits at the cost of others [11]. Indeed, in their recent meta-analysis spanning 50 species of arthropods and vertebrates, Macartney *et al*. [6] reported that seminal fluid was strongly condition dependent, while traits such as sperm quantity and quality were less consistently affected by diet restriction. Further, Macartney *et al*. [6] reported that the extent of condition dependence in ejaculate traits might be sensitive to the timing/developmental stage at which diet restriction is imposed, with a trend toward a stronger effect of juvenile diet limitation in some taxa and adult diet limitation in others (although these comparisons were not significant). These latter observations suggest that a more complete understanding of condition dependence in sexual traits will come from experiments that make direct comparisons of dietary restriction at different developmental stages, although only a limited number of such studies are currently available [12,13].

The guppy *Poecilia reticulata* is a live-bearing species of freshwater fish with some of the highest recorded rates of polyandry (female multiple mating) in any vertebrate [14]. Given the resulting importance of post-copulatory sexual selection in this species [15], guppies have been used extensively to explore resource dependence in ejaculate traits, with several studies reporting significant declines in a range of traits under dietary stress, including sperm motility, and the viability (% live), quantity and morphology of sperm [16–20]. Despite this strong evidence for condition-dependent expression of ejaculate traits, some studies have failed to replicate the general pattern of reduced trait expression under dietary restriction. Interestingly, some of the strongest effects of diet limitation on ejaculate quality, sperm production and sperm morphology have been reported in studies where experimental diets were administered to sexually immature juvenile males [17,19,20], while those same traits were largely unaffected in studies where diets were administered to fully mature males [9,10,18,21] (but see [16]). This raises the intriguing possibility that there is a critical phase of development during which dietary restriction has the strongest effect on these traits. However, this prediction has yet to be tested within a single controlled study of the same cohort of guppies.

Here, we assess the effects of dietary restriction when administered at different developmental stages on a range of post-copulatory sexually selected traits. Our analyses focus on dietary restriction at two critical phases of development that span sexual trait maturation in guppies [22,23]. Based on recent meta-analyses [6], and the findings cited above, we predicted that dietary restriction would have the strongest effects on ejaculate traits when administered to sexually immature males compared to adults. To test this prediction, our experiment incorporated three experimental groups that evaluated: (1) whether dietary restriction applied to juveniles compromises the ejaculate traits of newly mature males; (2) whether any observed effects of diet restriction on ejaculate traits in (1) are reversible when diet-stressed adults revert to an unrestricted diet; and (3) whether dietary restriction, when applied exclusively to mature (previously unstressed) males, has any additional effects on ejaculate traits beyond those predicted for groups (1) and (2).

## 2. Methods

### 2.1. Study population and its maintenance

The guppies used in this experiment were descendants of wild-caught fish captured from the Alligator Creek River in Queensland in 2006. The stock population from which experimental fish were taken was maintained in several large mixed-sex aquaria (84 × 45 × 36 cm^3^, filled to 29 cm; approx. 1 : 1 sex ratio; approx. 60–80 fish per tank) and fed a combination of dried food and *Artemia* nauplii under standard lighting conditions (12 : 12 light/dark cycle) at a constant temperature (26 ± 1°C). During the experimental treatment phase (see below), males were maintained individually in 2 l plastic tanks separated by paper dividers to avoid visual contact among test males. All test males had visual (but not direct) access via a non-permeable transparent glass barrier to a single stimulus female to ensure that they maintained sexual interest and sperm production during the treatment phase [24,25].

### 2.2. Experimental design

Experimental males were removed from their natal stock tanks as soon as they become morphologically distinguishable from females based on the differentiation of their anal fin into a gonopodium (intromittent organ), which occurs around six weeks of age [22]. At this point, males (*n* = 129 in total) were separated haphazardly into one of three groups designed to identify the critical ontogenetic period during which dietary restriction causes a decline in ejaculate quality. Following the methodology used for diet manipulation in previous studies on guppies (e.g. [10,16]), the groups were subdivided into two diet treatments: the ‘ad libitum’ treatment group was fed at a rate of approximately 4% of their body weight (maximum of 1.9 mg), while the ‘restricted’ treatment group was fed at a rate of approximately 2% of their body weight (maximum of 0.9 mg). Specifically, fish in the first and second groups described below, which were placed on diets at six weeks of age, were initially given approximately 75% of the full quantities in each diet. These were incrementally increased each week, until they were on their full quantities (1.9 mg and 0.9 mg, respectively) at 10 weeks of age, when they had reached adult body lengths but were still reproductively immature. Diets consisted of commercial dry food pellets (NutraKol Pty Ltd, Western Australia) administered once daily, 5 days a week.

The first group (*n* = 44), hereafter referred to as ‘**juvenile > short-term**’, was included as a baseline cohort to mimic prior studies that have reported resource-dependent reductions in sperm quality following dietary restriction when administered to maturing (sub-adult) males [16,19,20]. This **juvenile > short-term** group was divided evenly into those fed from approximately 6 weeks of age for eight weeks either a consistent *ad libitum* (high) or restricted (low) diet and tested immediately afterwards as newly matured adults at approximately 14 weeks of age. The second group (*n* = 43), referred to as ‘**juvenile > reversal**’, similarly applied experimental diets (high and low) to 6-week-old (maturing, sub-adult) males for eight weeks but included an additional subsequent ‘recovery’ phase of 8 weeks at a high diet for all the adult males before they were tested at approximately 22 weeks of age. This second group therefore allowed us to test whether the anticipated effects of dietary restriction during early development were reversable after males returned to a high diet. The third group (*n* = 42), termed ‘**adult treated**’, reversed this sequence of dietary regimens to determine whether dietary restriction applied exclusively at the adult stage caused a decline in sperm quality. Specifically, in this group six-week-old males were initially fed a high diet for 8 weeks before being assigned haphazardly into high- and low-quantity diet groups for another 8 weeks. This final group therefore enabled us to test whether dietary restriction applied exclusively to fully mature males caused a decline in sperm quality.

As described above, an inherent property of our experimental design is to compare responses to dietary treatments for fish that were tested at different age classes. Specifically, males from the **juvenile > short-term** treatment were tested at 14 weeks of age, while the remaining groups (**juvenile > reversal** and **adult treated**) were tested at 22 weeks of age. Thus, while we are specifically interested in the diet treatment and diet treatment-by-group effects (see Statistical analyses), any overall effect of age would be manifested as a main effect of group (e.g. younger fish might have lower sperm reserves/motility than older fish), which was not a primary focus of our analysis and indeed was not significant for sperm traits (see below). In the ensuing text, we refer to ‘group’ when describing the three broad developmental stage categories described here, and ‘diet treatment’ when referring to the high- and low-quantity diets that were applied within each of these distinct groups.

### 2.3. Sperm analyses and body size

Sperm traits were assayed immediately after males emerged from their respective feeding regimes. The males were first anaesthetized before being placed under a dissecting microscope on a glass slide for sperm retrieval. Briefly, the gonopodium was swung forward beyond 90° and 0.9% saline was added to the base of the gonopodium using a pipette. Light pressure was then applied to the male's abdomen to expel sperm [26]. Two aliquots, each comprising 2 μl and containing 3 sperm bundles, were then collected from the sperm pool and each placed in a separate chamber of a 12-well multi-test slide (MP Biomedicals, Aurora, OH, USA) previously coated with 1% polyvinyl alcohol to prevent sperm from sticking to the slide [27]. We then added 3 µl of 150 mmol l^−1^ KCl to each well to activate the sperm [28], which were immediately assayed for sperm motility (see below). The remainder of the ejaculate was collected from the slide and placed in an Eppendorf tube with a known volume of saline for subsequent sperm counts. Some fish (*n* = 7) produced no sperm and could not be assayed for any sperm traits. Others either did not have enough sperm bundles after collection for motility to obtain sperm counts (*n* = 3) or did not have enough motile sperm for computer-assisted sperm analysis (*n* = 5). Two fish did not have their weight recorded.

Sperm motility was estimated using computer-assisted sperm analyses (CASA; CEROS sperm tracker, Hamilton-Thorne Research, Beverly, MA, USA), which generated several measures of motility (see electronic supplementary material for full descriptions of these parameters). Briefly, these measures included the average path velocity (VAP), straight line velocity (VSL), curvilinear velocity (VCL), linearity (LIN), straightness (STR), beat cross frequency (BCF) and ‘wobble’ (WOB). As selection can target multiple sperm motility traits simultaneously due to correlations and interactions among component traits (e.g. [29]), we incorporated all CASA parameters into a principal component analysis (PCA; see below). However, we also augment these analyses with supplementary tests on VCL specifically, which describes the actual velocity of sperm cells across the track, as this parameter is known to predict competitive fertilization success in guppies [30,31]. The possible relationships between other specific CASA parameters and male reproductive fitness in guppies are presently unknown but given the strong genetic and phenotypic interrelationships among several of these parameters [31–33] we included them in our PCA for completeness. Sperm counts were estimated from the reserved ejaculate samples, which were vortexed for at least 10 s before being counted using an Improved Neubauer hemocytometer (Hirschmann Laborgeräte, Eberstadt, Germany). After sperm were stripped, each male was weighed to the nearest 0.01g using a Mettler Toledo Classic Plus (PB1502-S/FACT) balance and returned to post-experimental aquaria within the stock population.

### 2.4. Statistical analyses

Analyses were carried out in R v. 4.0.3 [34]. All data were tested for normality of residuals (from the linear models; see below) and homogeneity of variances using Shapiro–Wilk and Levene's tests, respectively, and transformed as appropriate to meet assumptions of parametric traits. For body weight, variances were homogeneous across both diets and groups, while residuals were marginally non-normal (Shapiro–Wilk: *W* = 0.98, *p* = 0.033). Attempts to transform body weight data did not improve the normality of residuals and therefore we ran the body weight analysis using raw data, noting that linear models are robust to departures from normality. Data for sperm counts were square root transformed to improve variances, which were homogeneous among treatments and groups after transformation. The PCA of sperm motility data generated two principal components (PCs) with eigenvalues > 1 (PC1: loaded predominantly by LIN and therefore a measure of path linearity; PC2: loaded predominantly by VCL and therefore a surrogate measure for sperm velocity; see electronic supplementary material). Both PCs exhibited normal residuals and equal variances among groups and treatments. The fixed effects of diet treatment, group (i.e. developmental stage at which diets were administered), and their interaction on each trait were tested as two-way analyses of variance (ANOVAs) using linear models in R. To test whether body size affected any of the measured ejaculate traits, we ran supplementary models for sperm count, PC1 and PC2 with body weight included as a fixed covariate, and all other parameters as described above.

## 3. Results

**Figure 1.**
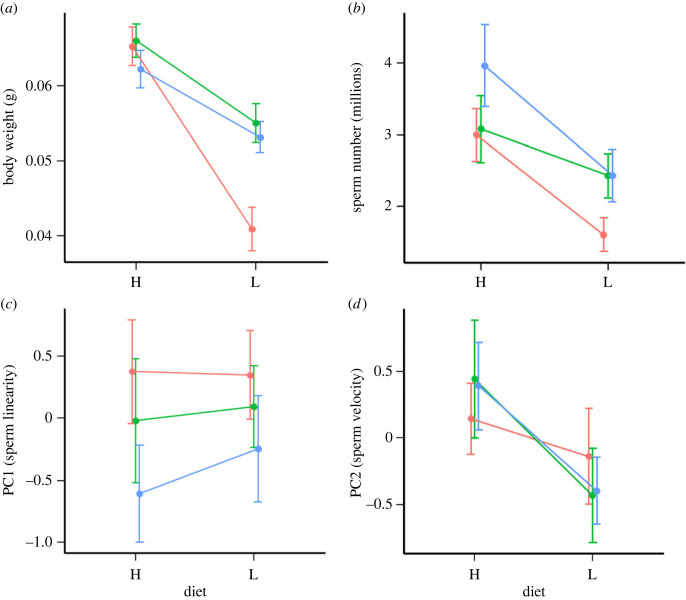
Means (± standard error) of (*a*) body weight, (*b*) sperm number, (*c*) sperm motility principal component 1 (sperm linearity), and (*d*) sperm motility principal component 2 (sperm velocity), at each level of diet treatment (H = high/ad libitum, L = low/restricted) and experimental group (red = **juvenile > short-term**, green = **juvenile > reversal**, blue = **adult treated**).

Our analysis revealed a highly significant effect of diet treatment, group (developmental stage) and their interaction on male body weight (table 1*a*); while dietary restriction impacted body weight in all three groups, the effect was by far the strongest when applied to **juvenile > short-term** fish that experienced dietary restriction prior to sexual maturity without opportunity for recovery (figure 1*a*). For sperm counts the effect of diet was highly significant; fish assigned to the restricted diet exhibited reductions in sperm counts compared to their ad libitum counterparts (table 1*b*); the effects of group and group-by-diet treatment interaction were not significant, although there appeared to be a trend toward particularly low sperm numbers for dietary-restricted males in the **juvenile > short-term** group (figure 1*b*). Finally, our analyses of sperm motility revealed significant effects of diet (with no group or interaction effect) on PC2 (sperm velocity) and no significant effect of diet, group or their interaction on PC1 (sperm linearity) (figure 1*c*,*d* and table 1*c*,*d*). These findings were supported by an analysis of curvilinear sperm velocity (VCL), which, in agreement with PC2, revealed a significant effect of diet treatment but no overall effects of group or group-by-treatment interaction (see electronic supplementary material). Further, inclusion of body weight as a covariate in the ejaculate trait models did not qualitatively alter any of the findings (see electronic supplementary material, table S3).

**Table 1.** Results from two-way analyses of variance (ANOVAs) testing the fixed effects of diet treatment, experimental group, and their interaction on (*a*) body weight, (*b*) sperm number, (*c*) sperm motility principal component 1 (equivalent to sperm linearity), and (*d*) sperm motility principal component 2 (equivalent to sperm velocity). Sperm number was square root transformed prior to analysis to meet the assumption of homogeneity of variances.

trait/factor	d.f.	*F*	*p*
(*a*) body weight			
diet	1	55.812	**<0.001**
group	2	4.498	**0.013**
diet × group	2	5.657	**0.005**
residuals	111		
(*b*) sperm number			
diet	1	13.358	**<0.001**
group	2	2.439	0.8092
diet × group	2	1.025	0.362
residuals	111		
(*c*) PC1 (sperm linearity)			
diet	1	0.102	0.750
group	2	1.864	0.160
diet × group	2	0.114	0.893
residuals	111		
(*d*) PC2 (sperm velocity)			
diet	1	5.440	**0.022**
group	2	0.002	0.998
diet × group	2	0.461	0.632
residuals	111

## 4. Discussion

This study demonstrates that dietary restriction in both sub-adult and adult age classes of guppy causes a significant decline in sperm numbers and sperm swimming velocity. Given the importance of both traits for reproductive success in guppies [30,31], these findings may have important implications for the expression of sexually selected traits in the wild given that natural populations typically experience seasonal variation in food availability [35]. Our findings thus emphasize the key role that resource dependency plays in post-copulatory sexual selection through its effects on ejaculates [6]. Further, our findings for body weight also suggest that newly mature males may sacrifice growth in order to maintain their investment in ejaculates, and only reinvest in somatic growth when they return to unrestricted diet conditions.

Our central finding was the overall consistency in the effects of sub-adult and adult dietary restriction on ejaculates (see also [12]), a finding that was unanticipated in the light of prior studies reporting far stronger resource-dependent responses in ejaculate traits when dietary restriction was confined to juvenile stages compared to those that manipulated diets in adults (see Introduction). Thus, we find little support for our primary hypothesis, although the non-significant trend for sperm numbers—which tended to be lower in **juvenile > short-term** fish under dietary restriction—was partially consistent with this expectation. One potential explanation for the discrepancy between the findings reported here for **adult treated** fish (i.e. those that experienced dietary restriction exclusively as newly matured adults) and prior work that also manipulated diet levels of adult age classes [9,10,18,21] is that previous studies tended to focus on adult males of unknown age taken from stock populations rather than newly matured males (14 weeks) as in the present study. In these previous studies, males were likely to be significantly older than 14 weeks used in the present study (some were known to be approximately 6 months old [18]) which suggests that the sensitivity of ejaculates to dietary stress may weaken as males continue to age beyond reaching sexual maturity. Nevertheless, the central prediction that there is a critical developmental ‘window’ during which ejaculates are sensitive to dietary stress is not supported by the present findings.

Our confirmation that dietary restriction imposed during juvenile life stages affects ejaculate traits in adults accords with a recent meta-analysis across taxonomic groups [6]. The general observation across taxa is that dietary limitation experienced by juveniles can have equivalent detrimental effects on ejaculates as dietary restriction in adults [6]; indeed in some taxa (e.g. mammals and arthropods) juvenile effects can be stronger (as was our original prediction for guppies). These general patterns suggest that the way in which juveniles mobilize their nutritional reserves during their development can have downstream effects on ejaculate production when they reach adulthood. Such effects may arise, for example, if dietary limitation in juveniles affects the development of the male reproductive organs in adults, subsequently altering their composition and/or size, as observed in the fruit fly *Drosophila melanogaster* [36] and the neriid fly *Telostylinus angusticollis* [37]. Such changes in the development of reproductive organs could feasibly have a permanent effect on ejaculates across a male's lifespan, which might explain why the effects of juvenile dietary restriction in our present study did not reverse when males were returned to a period of high diets. Understanding the physiological consequences of dietary restriction for reproductive traits would be an interesting direction for future research.

The results for body weight revealed far stronger effects of dietary limitation on body size than on ejaculate traits, which accords with the general pattern observed across mammals, arthropods and fishes (see [6]). Moreover, our finding that these effects were stronger for **juvenile > short-term** males than either the **juvenile > reversal** or **adult treated** groups is noteworthy because it suggests that the deleterious effects of dietary restriction endured during development for **juvenile > reversal** males were at least partially reversed when these fish returned to an ad libitum diet. Specifically, **juvenile > reversal** males that were assigned initially to the restricted diet were significantly larger than their **juvenile > short-term** (restricted) counterparts and indeed were similar to **adult treated** males who endured dietary restriction only after reaching sexual maturity. These findings suggest that males that have previously undergone dietary restriction may prioritize investment in growth upon reverting to a high diet. This latter finding may indicate that **juvenile > short-term** males sacrificed body mass to preserve sperm function, and only re-invested in body mass once food resources were restored. Notwithstanding the underlying proximate basis for these findings, there is clear evidence that dietary restriction has disproportionate effects on body weight (and therefore likely other condition-dependent traits) when experienced during early stages of development.

In conclusion, our study adds to an accumulating body of research showing that dietary restriction imposed at the juvenile stage can have downstream implications for ejaculate quality and sperm production in adults [12,13]. However, we also show that in the case of ejaculate traits, the effects of dietary restriction are similar, irrespective of whether dietary restriction is applied to juveniles or adults. We further show that ejaculates did not recover from the effects of dietary limitation following short-term reversals in dietary levels. By contrast, the results for body weight indicate that males can recover somatic growth when they return to ad libitum diets, suggesting that they may prioritize somatic growth when conditions improve, at least over the short term. Ideally, future studies will explore the proximate mechanisms linking dietary restriction during development and in adults to the decline in ejaculate trait expression, determine the extent to which these effects persist into adulthood, and explore their implications for reproductive fitness, ageing and survival.

## Data Availability

The data are provided in electronic supplementary material [38].
